# Instability Analysis and Free Volume Simulations of Shear Band Directions and Arrangements in Notched Metallic Glasses

**DOI:** 10.1038/srep34878

**Published:** 2016-10-10

**Authors:** Weidong Li, Yanfei Gao, Hongbin Bei

**Affiliations:** 1 Department of Materials Science and Engineering, University of Tennessee, Knoxville, TN 37996, USA; 2 Materials Science and Technology Division, Oak Ridge National Laboratory, Oak Ridge, TN 37831 , USA.

## Abstract

As a commonly used method to enhance the ductility in bulk metallic glasses (BMGs), the introduction of geometric constraints blocks and confines the propagation of the shear bands, reduces the degree of plastic strain on each shear band so that the catastrophic failure is prevented or delayed, and promotes the formation of multiple shear bands. The clustering of multiple shear bands near notches is often interpreted as the reason for improved ductility. Experimental works on the shear band arrangements in notched metallic glasses have been extensively carried out, but a systematic theoretical study is lacking. Using instability theory that predicts the onset of strain localization and the free-volume-based finite element simulations that predict the evolution of shear bands, this work reveals various categories of shear band arrangements in double edge notched BMGs with respect to the mode mixity of the applied stress fields. A mechanistic explanation is thus provided to a number of related experiments and especially the correlation between various types of shear bands and the stress state.

Similar to the concept of crack tip process zones, notch brittleness or ductility depends on the development of a clean process zone in the vicinity of the notch, which could be plastic deformation in metals or crack bridging or branching in composites, and a messy process zone right at the notch roots which are governed by damage processes on or below the microstructural length scales^1^,^2^. For examples, notches may not deteriorate the composite failure strength when the crack bridging zone (e.g., arising from fiber pull-out) is larger than the notch size. The study of notch sensitivity in bulk metallic glasses (BMGs), however, is much more complicated because of the localized deformation into shear bands, which can easily extend beyond the plastic zone estimated from continuum plasticity theory^3^,^4^,^5^. If the shear bands are not confined, either because the stress field is not decaying rapidly from the notch or due to the lack of geometric constraints, the resulting notch toughness will be low. Thus one commonly used approach of improving failure resistance and notch ductility is the introduction of geometric constraints that block and/or deflect the shear bands. Consequently, the degree of plastic strain on each shear band becomes low so that the transition from the shear band to a crack is delayed.

The clustering of multiple shear bands in the vicinity of notch roots has been investigated extensively in experiments^6^,^7^. The double edge notched samples under tensile condition have exhibited shear bands that connect the notches in Fig. 1(a)^8^,^9^,^10^, or radial shear bands that extend far from the notch roots in Fig. 1(b)^11^,^12^. It is worth noting that experiments in Sarac *et al*.^9^ were Mode I tests on Zr-based BMG films with a grid of pores at the gauge section, which is equivalent to the double edge notched sample since the shear bands are localized in the bridge between the holes. These two types of shear bands have not been found to co-exist, as understood by our schematic illustration in Fig. 1(c). The semi-circular shear bands that connect the neighboring notches actually lead to out-of-plane shear offset, and these shear bands will grow into the bulk in an inclined direction off the sample surface normal. The radial shear bands from the notch roots are believed to lead to surface ledges at the surface of the notch root. However, a mechanistic justification of such shear band arrangements has not been fully understood, and it has been suggested that these shear band patterns play an important role in understanding the notech sensitivity in recent experiments^7^,^8^,^13^. In addition to these Mode I (tension or compression) tests, Hsueh *et al*.^14^,^15^ performed combined compression/shear tests with various degrees of mode mixity (i.e., the ratio of Mode II to Mode I components). As shown in Fig. 2(a), when the applied loading condition is near the Mode II, two categories of shear bands can be found near the stress concentration sites – one being long, radial shear bands that extend almost across the entire sample, and the other being heavily curves shear bands that do not extend far from the edges. The latter becomes less prominent in Fig. 2(b) when the contribution of Mode II is reduced. Based on the measured load-displacement curves, Hsueh *et al*.^14^,^15^ developed a continuum plasticity model that considers the pressure effect in the yield surface, which however cannot address the importance of the non-uniform deformation fields and the shear band arrangements in these experiments.

This work presents a mechanistic analysis of the shear band arrangements in double edge notched BMGs, with a focus on the geometric constraints and the dependence on the mode mixity of the applied load. As opposed to the vast number of continuum plasticity simulations^3^,^4^,^8^,^11^,^12^,^13^,^14^,^15^, our analysis has a direct connection to the shear banding process, including their initiation from the material instability point of view and the shear band evolution from the free-volume-based constitutive model.

## Problem Definition

Consider a double edge notched specimen, as given in Fig. 3(a). Since the instability theory and the free volume model to be presented shortly do not involve any length scale, the deformation response is clearly governed by geometric parameters such as the ratios of notch root radius to sample width, notch depth to sample width, among many others. However, dimensions are still given in Fig. 3(a) for a convenient comparison to literature experiments^8^,^9^,^10^,^11^,^12^,^13^,^14^,^15^. The specimen has a dimension of 30 mm ×20 mm, with two notches of 5 mm in length and 0.5 mm in width that are located on the center of two side edges. The notch root is of a semi-circular shape with a radius of 0.25 mm. The top boundary is subjected to traction components of 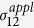 and 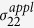. Similar to the fracture analysis, the mode mixity of the applied load is introduced as


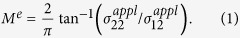


Mode-I (tension/compression) and Mode-II (shear) correspond to *M*^*e*^ = 1 and 0, respectively. Our simulations will be conducted with five representative values, i.e., *M*^*e*^ = 0, 0.25, 0.5, 0.75 and 1. Both the bottom edge and bottom right side edge of the sample are simply supported as given in Fig. 3(a). For the Mode II loading, additional displacement conditions are required for the top boundary to ensure a simple shear.

### Rudnicki-Rice instability theory

From the continuum mechanics point of view, the onset of strain localization results from the material instability and can be described by the general bifurcation theory^16^. The elastic-plastic deformation is initially homogeneous and stable. When reaching a critical condition (e.g., the loss of ellipticity in the constitutive law), the homogeneous deformation field becomes unstable, and the strain localizes in a narrow band while the surrounding material merely experiences elastic unloading. The exact formulation of the critical condition for strain localization is mathematically challenging, but Rudnicki and Rice^16^ derived a closed form representation for the shear band inclination angle. As depicted in Fig. 4, in the principal stress space, the shear band plane is parallel to the second principal stress component, and


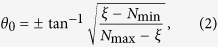


where 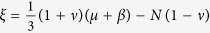, 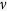 is Poisson’s ratio, *μ* is the coefficient of the internal friction (for generally pressure-sensitive materials like BMGs), *β* is the dilatancy factor (that describes the flow normality, i.e., associate flow if *μ* = *β*),


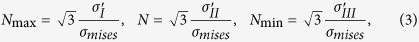


and 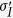, 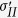, and 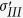 are the principal deviatoric stress components. To apply the Rudnicki-Rice instability theory, the elastic fields need to be obtained either from analytical approach or finite element simulations. When showing all the shear band directions on a regular grid, one can construct “streamlines” that are tangent to the shear band directions at any material point. Such a procedure to predict the shear band directions has been applied to a number of indentation problems with various geometric constraints such as the bonded interface micro-indentation test and the film/substrate system^17^,^18^,^19^.

### Free-volume based constitutive modeling

The above instability theory can only predict the onset of shear band, but not the subsequent evolution of shear bands and their interactions. A number of constitutive laws have been proposed in modeling the shear banding behavior of BMGs. These models share a common feature, that is, the stress-driven evolution of atomic structure leads to a strain softening behavior. If the structural recovery process is slow, the shear band formation will take place. The exact details in these models are of secondary importance if our interests are merely placed on the geometric arrangements of these shear bands. To this end, the free volume model^20^,^21^ is employed in our numerical simulations.

For a simple shear problem with applied shear stress *τ*, the shear strain rate is given by


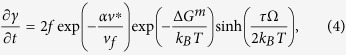


where *f* is the frequency of the atomic vibration, *α* is a geometric factor on the order of 1, v^*^ is the hard-sphere volume of an atom, *v*_*f*_ is the average free volume per atom, Δ*G*^*m*^ is the activation energy, Ω is the atomic volume, *k*_*B*_ is the Boltzmann constant, and *T* is the absolute temperature. The free volume, *v*_*f* _, can be regarded as an internal state variable that describes the structural information. Its evolution is governed by the competition between a stress-induced disordering process and a diffusional ordering process. The rate form is given by


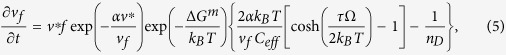


where *n*_*D*_ is the number of atomic jumps needed to annihilate a free volume equal to v^*^ and is usually taken to be 3–10, and the effective modulus is *C*_*eff*_ = *E*/3(1-*v*) with *E* being the Young’s modulus.

The above equations form the basis for a constitutive law, and they can be generalized into the multiaxial stress state by using the Mises stress component. This generalized model has been implemented in ABAQUS, a commercial finite element software, through the user-defined material (UMAT) subroutine^22^, and has been applied to study a number of indentation problems^18^,^23^. In our finite element simulations for the problem defined in Fig. 3, we choose the following constitutive parameters: *E*/*σ*_0_ = 240, *v* = 0.333, *n*_*D*_ = 3, *α* = 0.15, and *σ*_0_ = *k*_*B*_*T*/Ω is the reference stress that is used to normalize the stress tensor and modulus. It should be noted that this constitutive law does not contain a length scale, so that the simulated shear bands will depend on the mesh size in terms of the shear band width and also on the directional alignment of the elements since the shear bands may spuriously grow into regimes with uniform mesh density when the stress gradient is low^22^. Consequently, our finite element mesh is designed in Fig. 3 to minimize the mesh size and the directional alignment of these elements. Another drawback of this model is that the initiation of shear bands is driven by the stress concentration, where the free volume increases rapidly according to Eq. (5). Recent experiments have found that the shear banding process critically depends on the initial atomic structure, and particularly its fluctuation^24^,^25^,^26^. As a consequence, the strain field is found to fluctuate in early stage of deformation and sometimes even below the macroscopic yield point. These observations cannot be simulated in the above free volume model again because of the lack of a length scale.

## Results and Discussion

From the free-volume-based finite element simulations, the applied traction versus the engineering strain curves are plotted in Fig. 5 for the un-notched sample under plane-strain tension and the notched samples with a number of mode mixity values. The un-notched sample deforms essentially as a single element, and its peak value and the subsequent softening rate are mostly governed by the loading rate and constitutive parameters in the free volume model in Eqs (4) and (5). It should be noted that if a failure model is added to the constitutive law (e.g., one candidate model being the cavity nucleation and growth in the shear band), the stress-strain curves will exhibit sharp stress drops after the peak stress.

Under the Mode II loading condition (*M*^*e*^ = 0), the predicted shear bands from the Rudnicki-Rice theory consist of two categories in Fig. 6(a). The dominant family of shear bands, as shown by black curves, emanates from the notch roots and traverses almost through the entire sample. The other family (red shear bands) does not extend far from the notch root. Note that these shear bands are predicted from a number of seed points near the notch root. The termination of red shear bands simply suggests a change of stress state ahead of these shear bands, thus changing the directions of the principal stress components. The average inclination angle from the dominant shear bands is found to be about 14°. In the free-volume-based finite element simulations, a shear band first appears at the left notch and grows in radial direction, followed by the initiation and growth of two shear bands from the right notch. The contour plots represent the free volume field (SDV1), which indicates the locations of the shear bands. Shear bands at the right notch are different in the instability theory and free-volume model, because the instability theory is based on the stress field before shear band initiation, while the initiation and growth of first several shear bands will change the stress field. For example, Singh *et al*.^27^,^28^ introduced radial weak zones near a crack tip and found the initiation of cavitation failure in these weak zones was significantly promoted due to the interactions of these weak zones.

Under mixed mode loading conditions, *M*^*e*^ = 0.25, 0.5 and 0.75 in Figs 7, 8 and 9 respectively, a new family of shear bands (blue) emerge and gradually extend further into the middle of the sample when the mode mixity has an increase degree of Mode I. Eventually, the Mode I loading in Fig. 10 sees the symmetric arrangement of black and blue families of shear bands. The red family of shear bands in Figs 7, 8, 9 is always contained near the notch roots because the change of the stress state does not permit them to extend far. The inclination angle of the black family of shear bands increases with respect to *M*^*e*^, reaching about 45° when approaching Mode I loading condition in Fig. 10. The free-volume-based finite element simulations largely agree with predictions from the instability theory in the following: (i) the inclination angles of the dominant shear bands, and (ii) a number of short shear bands that do not extend far from the notch roots. But as we have seen in Fig. 6, the major shear bands that appear in early stage will change the stress state near the notch roots, so the secondary shear bands may differ from the predictions by the instability theory.

**Figure 1:**
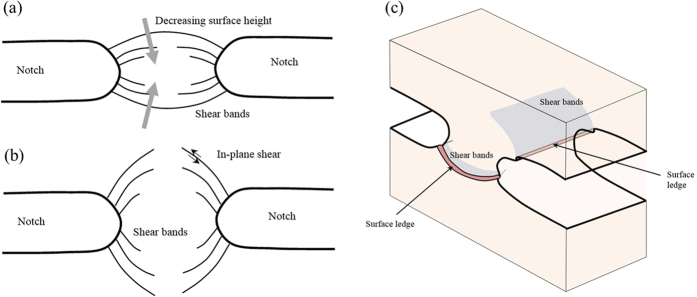
Schematic illustration of the two types of shear band arrangements commonly observed in experiments. (**a**) Semi-circular shear bands are found to connect the two notches, and these shear bands lead to out-of-plane surface steps^8^,^9^,^10^. (**b**) Shear bands emanating from the notch roots and leading to in-plane shear deformation^11^,^12^ (**c**) A three-dimensional illustration of the surface ledges/steps caused by these two types of shear bands.

Under the Mode I loading condition (*M*^*e*^ = 1), the Rudnicki-Rice theory predicts symmetric arrangements of radial shear bands from the notch roots, with an inclination angle of about 41° in Fig. 10(a). The free-volume-based finite element simulations give an angle of about 47°. Note that the boundary conditions in Fig. 3(a) were adopted in the mixed mode loading conditions, and also for Mode I condition for the sake of consistency. These applied boundary conditions will break the symmetry in the *x* axis, so that only the black family of shear bands is observed in Fig. 10(b–d). Experimental observations routinely observe these shear bands^11^,^12^, also as shown in Fig. 1(a,c). Zhao *et al*.^10^ found an inclination angle of 40~41° in their experiments, which agree perfectly with our predictions in Fig. 10.

A new family of shear bands, as marked green, is found in the Mode I condition in Fig. 10(a). According to the Rudnicki-Rice model in Fig. 4, the shear band plane parallel to the second principal stress. Therefore, the principal stress directions obtained from the Mode I elastic simulation in Fig. 11 suggest the presence of the out-of-plane shear in the region enclosed by the dashed curves but in-plane shear in the remaining area. The predicted trajectories in green correspond to the out-of-plane shear bands as depicted in Fig. 1(a). In addition, we caution that our predictions here are based on plane-strain condition. Experimental observations^8^,^9^,^10^ were conducted on the surface, where the stress field is close to a plane-stress condition. As shown in Fig. 1(c), the traction-free condition on the front surface will further facilitate the initiation of these green shear bands. For thin samples, these green shear bands will run through the thickness direction and leave inclined steps (on the sample front/back surfaces) when viewing from horizontal direction. For thick samples, their importance diminishes, and the dominant shear bands are those running in radial direction from the notch roots, resulting in steps/ledges at the notch root surface. The free volume simulation is unable to predict these out-of-plane shear bands because of the plane strain condition in the finite element simualtions. It should aslo be noted that a recent Mode-I tensile test of notched samples found strain hardening as a result of stress-driven diffusional relaxation and densification of the metallic glass^29^. It is probably related to the more complicated dependence of free volume field on the multiaxial stress state, while the model adopted in this work only assumes *J*_2_ plasticity and does not include a long-range diffusional mechanism. Further investigations need to be conducted on the relationship between the constitutive model and such an observed strain hardening behavior^29^.

**Figure 2:**
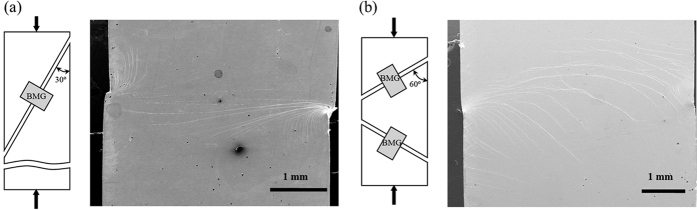
Combined compression-shear tests on bulk metallic glasses lead to complex shear band arrangements. (**a**) With a sharp inclined angle, the SEM image exhibits heavily inclined shear bands near the stress concentration sites. (**b**) With a low inclined angle, the shear bands tend to align with the direction of the applied shear force. Micrographs are adapted from refs 14 and 15.

**Figure 3:**
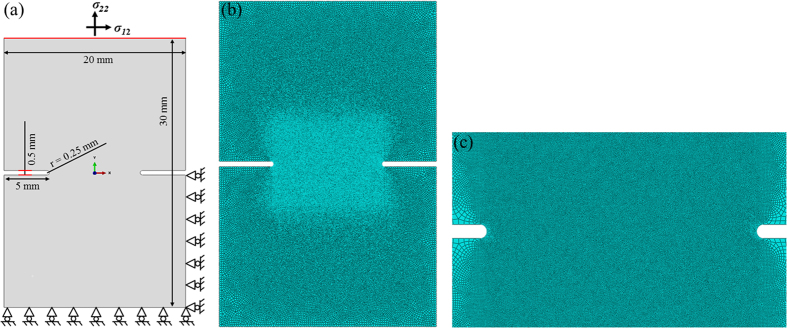
Model setup used for both the Rudnicki-Rice instability analysis and the free volume modeling. (**a**) The double-edge notched sample for our numerical simulations. The mode mixity is controlled by varying the ratio of the applied tractions, 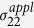 and 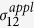, on the top boundary. (**b**) Finite element mesh used in both elastic stress analysis and free-volume-based shear band simulations. Since the free volume model shows mesh size dependence, the mesh density is chosen to be high throughout the highly stressed regime and also the mesh design minimizes its directional alignment. (**c**) Enlarged view near the notch root.

**Figure 4:**
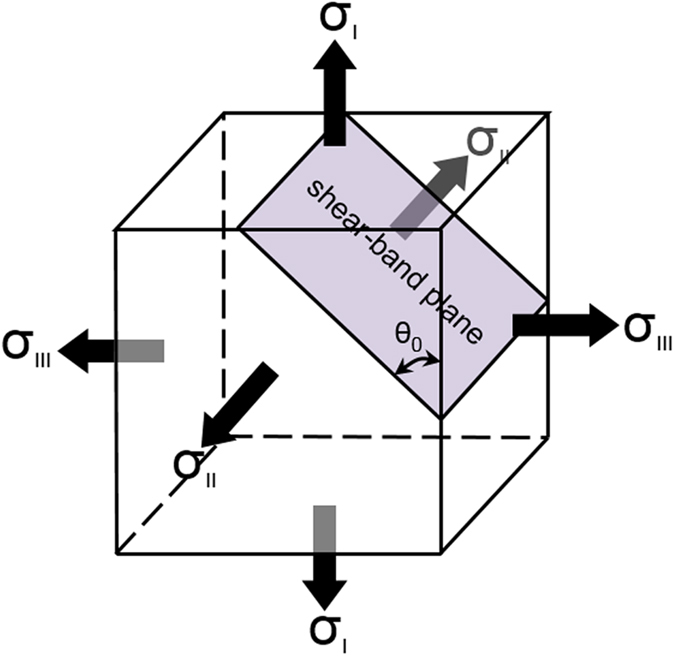
Schematic illustration of the shear-band plane predicted by the Rudnicki-Rice instability theory in the principal stress space. According to the Rudnicki-Rice instability analysis, the strain field will localize into a narrow band, with its normal in the plane spanned by *σ*_1_ and *σ*_*III*_ which are the largest and smallest principal stress components, respectively. In other words, the shear-band plane is parallel to the second principal stress component, *σ*_*II*_. Note that if *θ*_0_ = 45°, the shear band follows the maximum shear stress plane, which however is generally not true.

**Figure 5:**
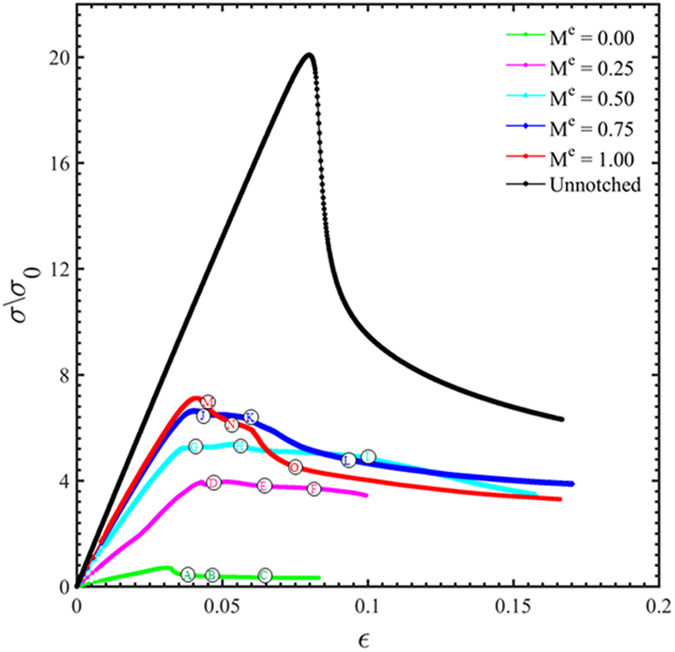
The applied stress versus the engineering strain of the notched sample. 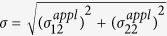
, 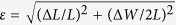, and *L* and *W* are sample length and width, respectively.

**Figure 6:**
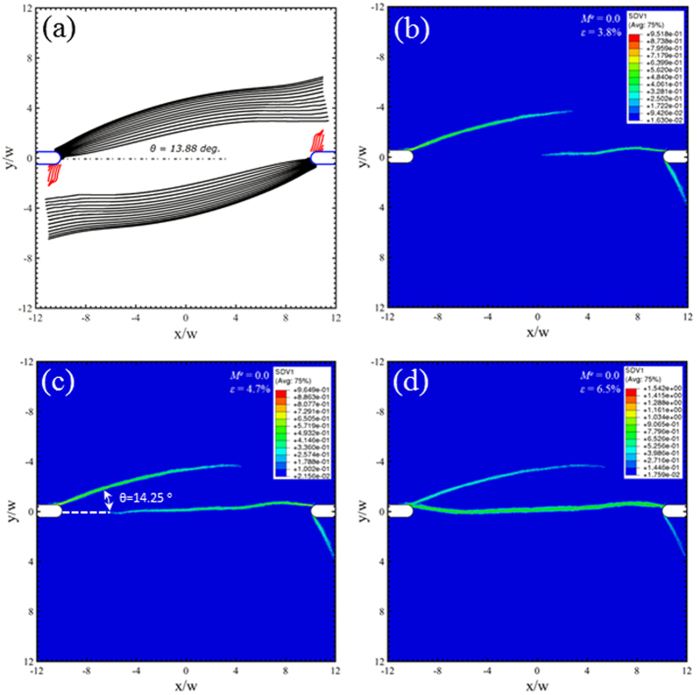
Predicted shear band configurations at *M*^*e*^ = 0, corresponding to the Mode-II shear. Prediction from (**a**) the instability theory, and (**b–d**) the free-volume-based constitutive simulations at three different strain stages, which are marked as “A”, “B”, and “C” on Fig. 5.

**Figure 7:**
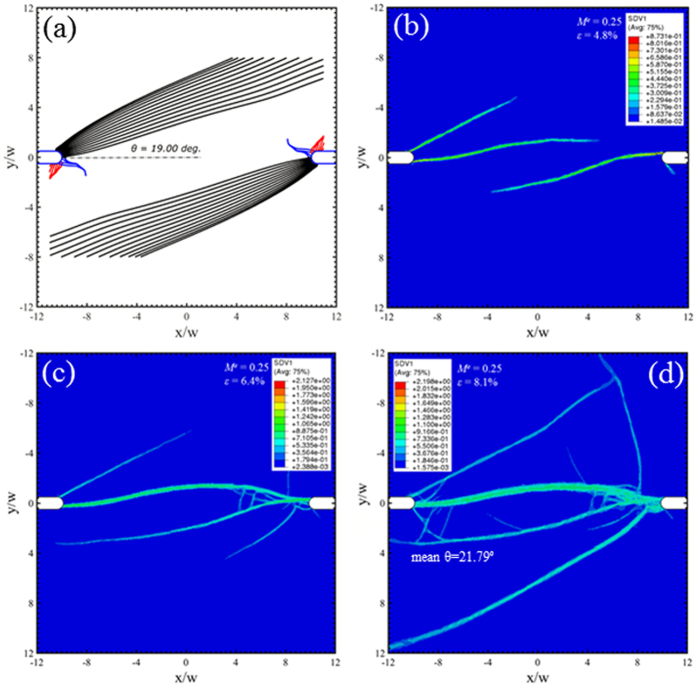
Predicted shear band configurations at *M*^*e*^ = 0.25. Prediction from (**a**) the instability theory, and (**b–d**) the free-volume-based constitutive simulations at three different strain stages, which are marked as “D”, “E”, and “F” on Fig. 5.

**Figure 8:**
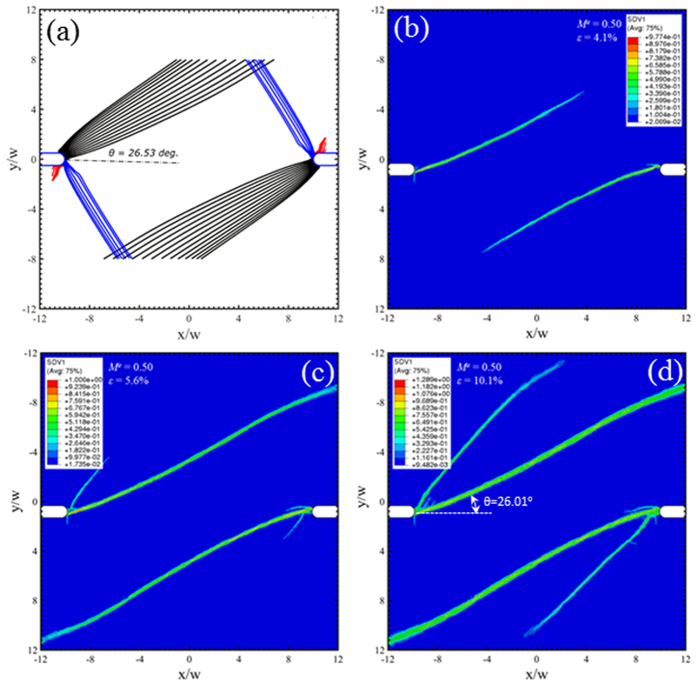
Predicted shear band configurations at *M*^*e*^ = 0.5. Prediction from (**a**) the instability theory, and (**b–d**) the free-volume-based constitutive simulations at three different strain stages, which are marked as “G”, “H”, and “I” on Fig. 5.

**Figure 9:**
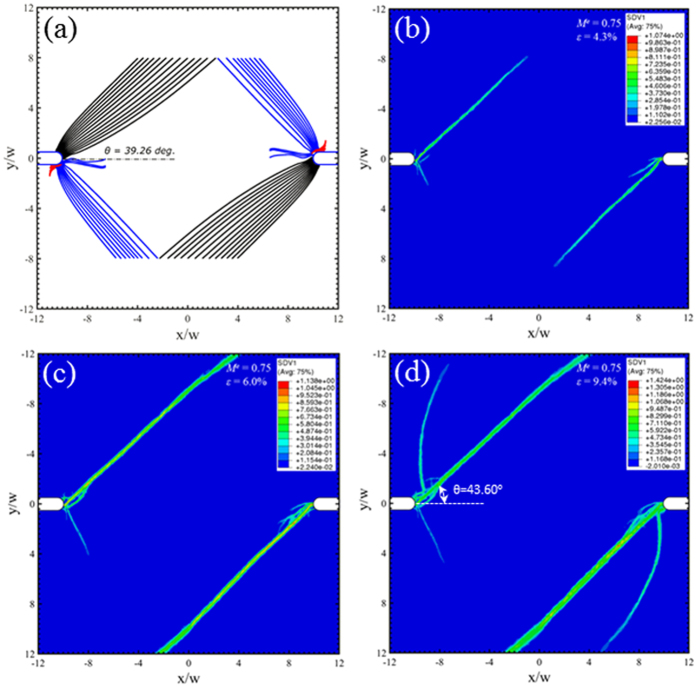
Predicted shear band configurations at *M*^*e*^ = 0.75. Prediction from (**a**) the instability theory, and (**b–d**) the free-volume-based constitutive simulations at three different strain stages, which are marked as “J”, “K”, and “L” on Fig. 5.

**Figure 10:**
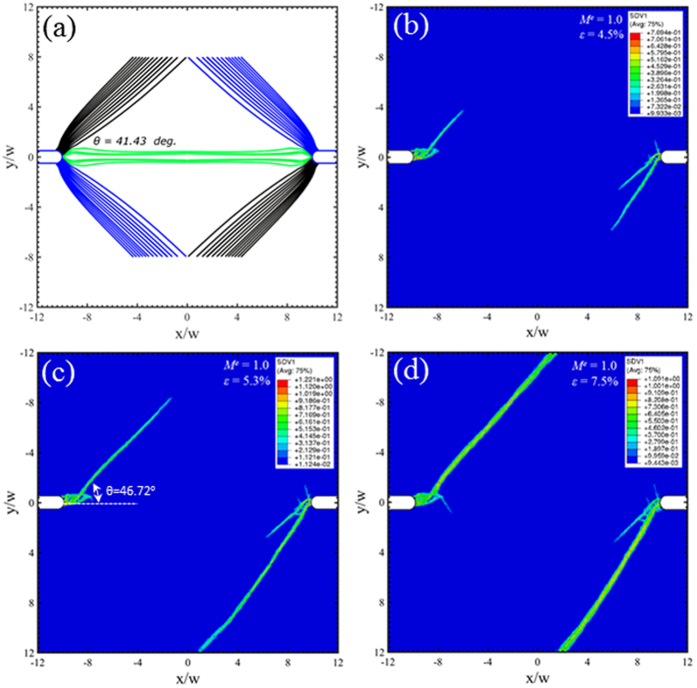
Predicted shear band configurations at *M*^*e*^ = 1.0, corresponding to the Mode-I tension. Prediction from (**a**) the instability theory, and (**b–d**) the free-volume-based constitutive simulations at three different strain stages, which are marked as “M”, “N”, and “O” on Fig. 5.

**Figure 11:**
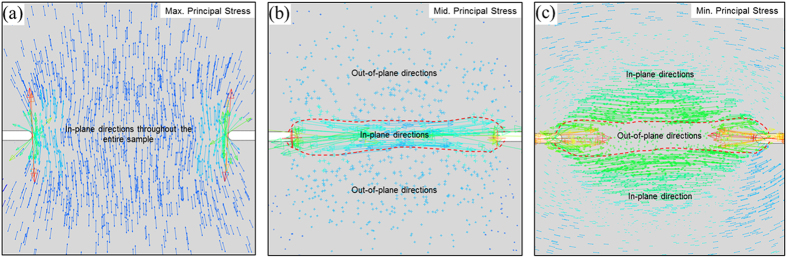
Direction vectors of the three principal stress components projected on the (*x*_1_, *x*_2_) plane for the Mode-I loading condition as in Fig. 10(a) *σ*_*I*_, (b) *σ*_*II*_, and (c) *σ*_*III*_,. The direction of *σ*_*II*_, in the bridging zone between the two notches differs from that elsewhere, indicating a change of shear band direction according to the Rudnicki-Rice prediction in Fig. 4.

**Figure 12:**
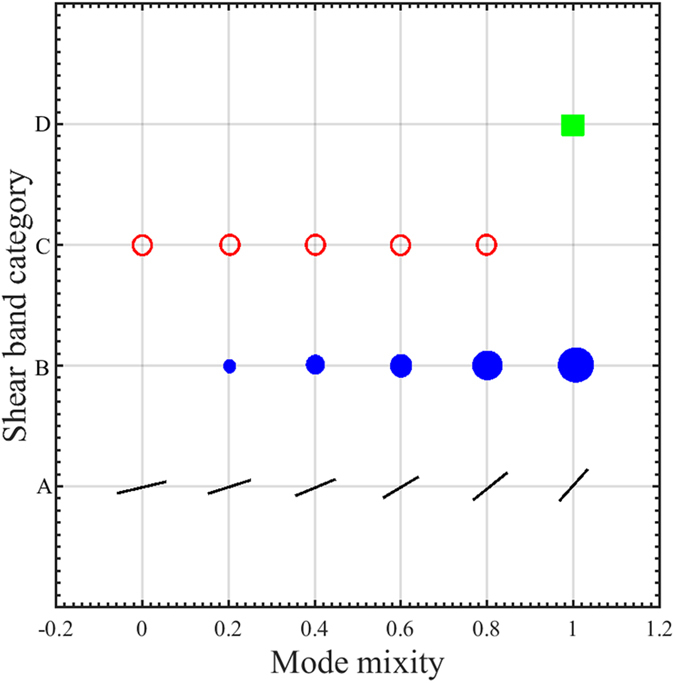
Categories of shear band arrangements as summarized from Figs 6, 7, 8, 9, 10. Category A represents the dominant, radial shear bands whose inclination angles come greater with the increase of the mode mixity. Category B represents the secondary radial shear bands, of which the extent of shear-band zone and their inclinations angles increase as the mode mixity increases. The shear bands in Category C cluster near the notch root and do not extend far beyond a length larger than the root radius. Category D is unique for the Mode I condition.

**Figure 13:**
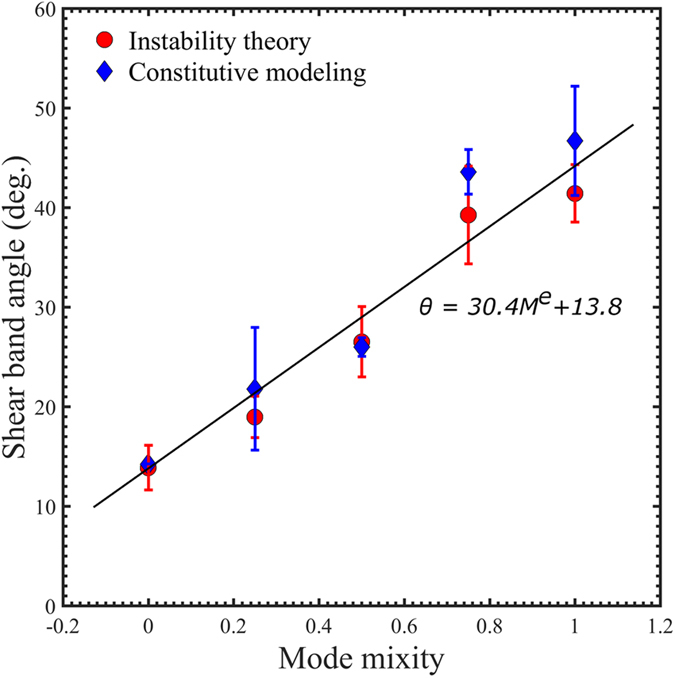
Variation of the inclination angle of the dominant, radial shear bands (Category A) with respect to the mode mixity. Red circles and blue diamonds are from the instability theory and constitutive simulations, respectively. Data correspond to those in Table 1.

Predictions in Figs 5, 6, 7, 8, 9, 10 are summarized in Fig. 12. First, we note that the radial (black) shear bands dominate over the entire span of the mode mixity, while the secondary (blue) shear bands gains a more important role with the increase of the mode mixity. Referring back to the experiments in Fig. 2, the loading conditions leads to a shift of mode mixity from a low value (near Mode II) to a high value (near Mode I). Our predictions can successfully explain the observed shear band arrangements, in which the dominant family of shear bands extends throughout the sample but the secondary shear bands are confined near the stress concentration sites. These results also resemble the localized bands found in concretes^30^, which clearly demonstrate the role played by the dependence of the stress state on the loading conditions. Second, with the increase of the mode mixity from Mode II to Mode I, the angle of the major shear bands increases from about 14° to about 41°. A linear fitting to the data summarized in Table 1 gives


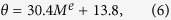


as plotted in Fig. 13. Assume that the characteristic time associated with the propagation of individual shear bands scales with the ratio of the shear-band traveling distance to the shear band speed. The latter has been found to be insensitive to the normal stress on the shear band^31^. Using an estimate of 100 m/s, it is anticipated that the shear-band-propagation time scale is about 0.1 ms in Mode II loading and about 0.07 ms in Mode I loading condition. These time scales should correspond to the stick-slip behavior, or called flow serrations, on the stress-strain curves observed experimentally^32^.

## Conclusion and Summary

In contrast to the vast number of literature works that are based on continuum plasticity model and simulations, we predict the initiation of shear bands from the Rudnicki-Rice instability theory, and simulate the shear banding process from the free-volume-based finite element method. A systematic study of the shear band orientations and arrangements is presented for double edge notched BMGs with various loading conditions. Our analysis enables us to develop a mechanistic interpretation of the conditions for semi-circular and radial shears in Fig. 1, as commonly observed in experiments, and also our simulation results rationalize the shear band arrangements under mixed mode loading conditions in Fig. 2.

## Additional Information

**How to cite this article**: Li, W. *et al*. Instability Analysis and Free Volume Simulations of Shear Band Directions and Arrangements in Notched Metallic Glasses. *Sci. Rep*. **6**, 34878; doi: 10.1038/srep34878 (2016).

## Figures and Tables

**Table 1:** Shear band inclination angle, as described by *θ* in Figs 6, 7, 8, 9, 10, 11, 12, with respect to the degree of mode mixity.

Mode mixity		0	0.25	0.5	0.75	1.0
Shear-band angle from instability theory (in degrees)	Mean value	13.88	19.00	26.53	39.26	41.43
	STD	2.25	2.08	3.53	4.92	2.89
Shear-band angle from constitutive modeling (in degrees)	Mean value	14.25	21.79	26.01	43.60	46.72
	STD	n/a	6.16	0.93	2.45	5.48

The average values and the standard deviations (STDs) compared between the Rudnicki-Rice instability theory and the free-volume-based constitutive modeling.
